# KSHV Manipulates Notch Signaling by DLL4 and JAG1 to Alter Cell Cycle Genes in Lymphatic Endothelia

**DOI:** 10.1371/journal.ppat.1000616

**Published:** 2009-10-09

**Authors:** Victoria Emuss, Dimitrios Lagos, Arnold Pizzey, Fiona Gratrix, Stephen R. Henderson, Chris Boshoff

**Affiliations:** 1 Cancer Research UK Viral Oncology Group, UCL Cancer Institute, University College London, London, United Kingdom; 2 Research Department of Haematology, UCL Cancer Institute, University College London, London, United Kingdom; Emory University, United States of America

## Abstract

Increased expression of Notch signaling pathway components is observed in Kaposi sarcoma (KS) but the mechanism underlying the manipulation of the canonical Notch pathway by the causative agent of KS, Kaposi sarcoma herpesvirus (KSHV), has not been fully elucidated. Here, we describe the mechanism through which KSHV directly modulates the expression of the Notch ligands JAG1 and DLL4 in lymphatic endothelial cells. Expression of KSHV-encoded vFLIP induces JAG1 through an NFκB-dependent mechanism, while vGPCR upregulates DLL4 through a mechanism dependent on ERK. Both vFLIP and vGPCR instigate functional Notch signalling through NOTCH4. Gene expression profiling showed that JAG1- or DLL4-stimulated signaling results in the suppression of genes associated with the cell cycle in adjacent lymphatic endothelial cells, indicating a role for Notch signaling in inducing cellular quiescence in these cells. Upregulation of JAG1 and DLL4 by KSHV could therefore alter the expression of cell cycle components in neighbouring uninfected cells during latent and lytic phases of viral infection, influencing cellular quiescence and plasticity. In addition, differences in signaling potency between these ligands suggest a possible complementary role for JAG1 and DLL4 in the context of KS.

## Author Summary

Kaposi sarcoma herpesvirus (KSHV) is a tumour virus associated with Kaposi sarcoma (KS). Most KS tumor cells are latently infected with the virus, while a small number are lytically infected and produce KSHV. The Notch signaling pathway is highly conserved and important in development and disease. Classical activation of this pathway occurs through direct interaction between ligands and receptors bound to the surface of adjacent cells and influences gene expression in cells receiving the signal. KS tumour cells express Notch pathway components and are sensitive to inhibition of Notch signaling, suggesting this pathway may be important in the development of KS; however, no mechanism behind the classical activation of Notch by KSHV has been established. We describe the molecular mechanisms through which KSHV hijacks the Notch signaling pathway by directly increasing the expression of two Notch ligands (JAG1 and DLL4) through two KSHV genes expressed during latent and lytic infection, respectively. We show the effect of JAG1- and DLL4-stimulated signaling on gene expression in adjacent cells and show that both ligands affect cell cycle-associated genes and may co-operate to permit functional signaling in the context of both latent and lytic infection.

## Introduction

The Notch pathway is an evolutionarily conserved signaling mechanism that transduces signals between adjacent cells and has an established role in cell fate determination during development, tissue homeostasis and stem cell maintenance [1],[2]. The Notch receptors (NOTCH1–NOTCH4) and ligands (JAG1, JAG2, DLL1, DLL3, and DLL4) are membrane-bound proteins that associate through their extracellular domains. Receptor-ligand interaction stimulates sequential proteolytic cleavage events at the receptor that release the intracellular domain (ICD) for translocation to the nucleus of the receiving cell. The ICD contributes to a ternary complex, involving the transcription factor CSL (CBF-1, Su(H), Lag-1), and upregulates transcription of target genes, primarily members of the HES and HEY families of transcriptional repressors [3]. The outcome of Notch signaling is cell-type dependent [4]–[6] and this pathway has essential roles during physiological and pathological angiogenesis [7].

NOTCH1, NOTCH4, JAG1 and DLL4 are expressed on vascular endothelium. New vessel “tip” cells form the guiding cells of endothelial sprouts and Notch signaling is essential for the specification of these cells. Ligand expression confers the tip phenotype and suppresses it in neighbouring receiving cells under physiological (DLL4) and pathological (JAG1) conditions [8]–[11]. Distinct spatial expression of DLL4 and JAG1 in normal developing vasculature suggests that ligand-specific outcomes of Notch signaling are required for normal development [12],[13]. Cells adjacent to the tip cells form the stalk of the vessel and are subject to quiescent growth arrest. Notch signaling is also implicated in the maintenance of a reversible, quiescent state in stem cell progenitors [14],[15] and is associated with growth arrest in a number of systems through manipulation of cell cycle components including minichromosome maintenance (MCM) proteins and cyclin dependent kinase inhibitors (CDKIs) [16]–[19].

Kaposi sarcoma herpesvirus (KSHV, also called HHV-8) is an oncogenic γ-herpesvirus that is the etiological agent of Kaposi sarcoma (KS), a neoplasm of lymphatic endothelial cells (LEC) [20]. KSHV is also associated with lymphoproliferations such as multicentric Castleman's disease (MCD) [21]. KS is an angioproliferative disease composed of sheets of spindle cells (the KS tumour cells), an inflammatory infiltrate and abnormal slit-like blood vessels. All KS spindle cells are infected by KSHV [22]. During the establishment of host infection, two phases of viral infection exist: latent and lytic. The majority of spindle cells are latently infected and express a limited number of viral genes including the viral FLICE inhibitory protein (vFLIP); productive (lytic) viral infection is associated with expression of an increased number of viral genes including the multifunctional viral G protein-coupled receptor (vGPCR) [23].

KS lesions express elevated levels of Notch signaling components and experimental lesions appear sensitive to inhibition of this pathway [24],[25]. The KSHV ORF50 gene product, RTA, has been shown to induce expression of HEY1 during lytic reactivation of the virus [26]–[28], but a mechanism through which KSHV alters the expression of the other Notch-associated proteins, specifically during latency, has not been described. Here we show that KSHV specifically increases the expression of the Notch ligands JAG1 and DLL4 and the receptor NOTCH4 in LEC. The increase in JAG1 and DLL4 is attributable to the viral genes vFLIP and vGPCR, through mechanisms dependent on the NFκB and ERK pathways respectively. We demonstrate that JAG1 and DLL4 stimulate Notch signaling in adjacent LEC and alter the expression of cell cycle-associated genes. The suppression of a number of these genes is observed in LEC adjacent to vFLIP- and vGPCR-expressing cells and during KSHV infection of LEC; the effect of Notch on cell cycle components could offer a growth advantage to infected cells during the pathogenesis of KS. These data also suggest that DLL4 and JAG1 may have a similar role during sprouting lymphangiogenesis as has been observed in blood vessel endothelial cells during angiogenesis, where Notch induces quiescence in developing vascular sprouts.

## Results

### KSHV infection of LEC increases the expression of specific components of the Notch signaling pathway and activates canonical Notch signaling

**Figure 1:**
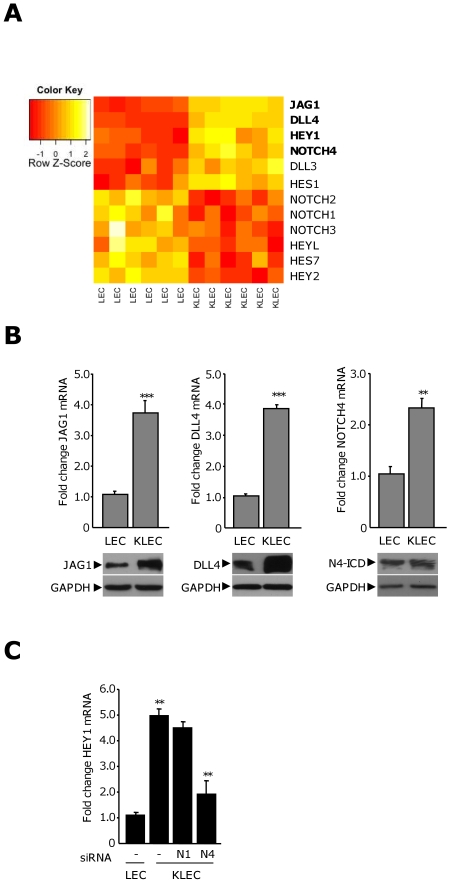
KSHV alters the expression of core Notch components in LEC and activates canonical Notch signalling. (A) Heatmap representing the most significant relative changes in gene expression of Notch pathway components in LEC following KSHV infection (q value ≤ 0.05). Gene names in bold indicate genes significantly altered in the context of a list of 79 angiogenic genes. Genes are ordered by magnitude of fold change. Original GEM data from [29]. Red and yellow denote low and high expression respectively. (B) Graphs of the average fold change in the indicated mRNA in LEC and KLEC from at least three independent experiments. **, *P*<0.01, ***, *P*<0.001, with respect to LEC. Representative corresponding protein levels shown below each graph. (C) HEY1 mRNA levels in LEC and KLEC transfected with non-silencing siRNA (−) or siRNA targeting NOTCH1 (N1) or NOTCH4 (N4). Columns are the average fold change from three independent experiments. **, *P*<0.01, with respect to LEC or KLEC transfected with a non-silencing control.

KS has been shown to be sensitive to γ-secretase inhibition in murine models [24],[25]. We have previously described the transcriptional signature of KSHV-infected LEC (KLEC) [29] and therefore analysed these data with respect to the expression of the core components of the Notch signaling pathway including HES and HEY Notch targets (Figure 1A). This analysis indicated significant changes in the expression of specific members of the pathway at the mRNA level following KSHV infection (false discovery rate threshold q≤0.005; Figure 1A). Significant increase in expression was restricted to three ligands (DLL4, JAG1 and DLL3), the HES1 and HEY1 targets and the NOTCH4 receptor. The expression of all other Notch receptors was significantly decreased along with the remaining Notch target genes analysed. The expression of an additional Notch target, HES5, and the Notch ligand DLL1 were not significantly altered.

The established role of Notch signaling through these components during angiogenesis prompted us to consider these data with respect to a list of 79 angiogenesis-associated genes that are significantly altered in KLEC [30]. Notch ligands, JAG1 and DLL4, the NOTCH4 receptor and the Notch target, HEY1 were among the most highly upregulated (in bold in Figure 1A). The gene expression microarray (GEM) data for these components were validated at the mRNA and protein levels (Figure 1B). Western blotting for NOTCH4 indicated a band at ∼70 kDa, the predicted size of the NOTCH4 intracellular domain (N4-ICD); no band corresponding to the full-length protein was observed. This indicated that the increased NOTCH4 could be involved in active Notch signaling in LEC.

Notch pathway activation has been described in cells from plaque stage KS lesions [25], suggesting that this pathway has a role in latent infection. NOTCH4 was the most significantly upregulated receptor in the context of primary KSHV infection. We therefore investigated whether the upregulation of HEY1 in KLEC was dependent on the expression of this receptor (Figure 1C). LEC were transfected with siRNA against the NOTCH4 receptor, and NOTCH1 as a control, to achieve knock-down of the corresponding mRNA of 50% and 40% respectively (Figure S1). KSHV induced a reproducible five-fold increase in HEY1 expression. This increase was unaffected by the knock-down of NOTCH1, but was reduced by approximately 60% in the presence of NOTCH4 siRNA (Figure 1C). These data suggest KSHV increases HEY1 levels in LEC through a mechanism dependent on the NOTCH4 receptor. They also indicate that there is no functional redundancy between NOTCH1 and NOTCH4 in this system as the maintained expression of NOTCH1 was not sufficient to rescue HEY1 levels in the presence of NOTCH4 knock-down. HEY1 levels were not reduced to baseline levels in the presence of NOTCH4 siRNA; this may be attributed to a receptor-independent induction of HEY1 as a consequence of the expression of low levels of KSHV ORF50 during primary infection [28],[31], or due to incomplete knock-down of NOTCH4.

### vFLIP increases levels of JAG1 through an NFκB-dependent mechanism and instigates Notch signaling through NOTCH4

**Figure 2:**
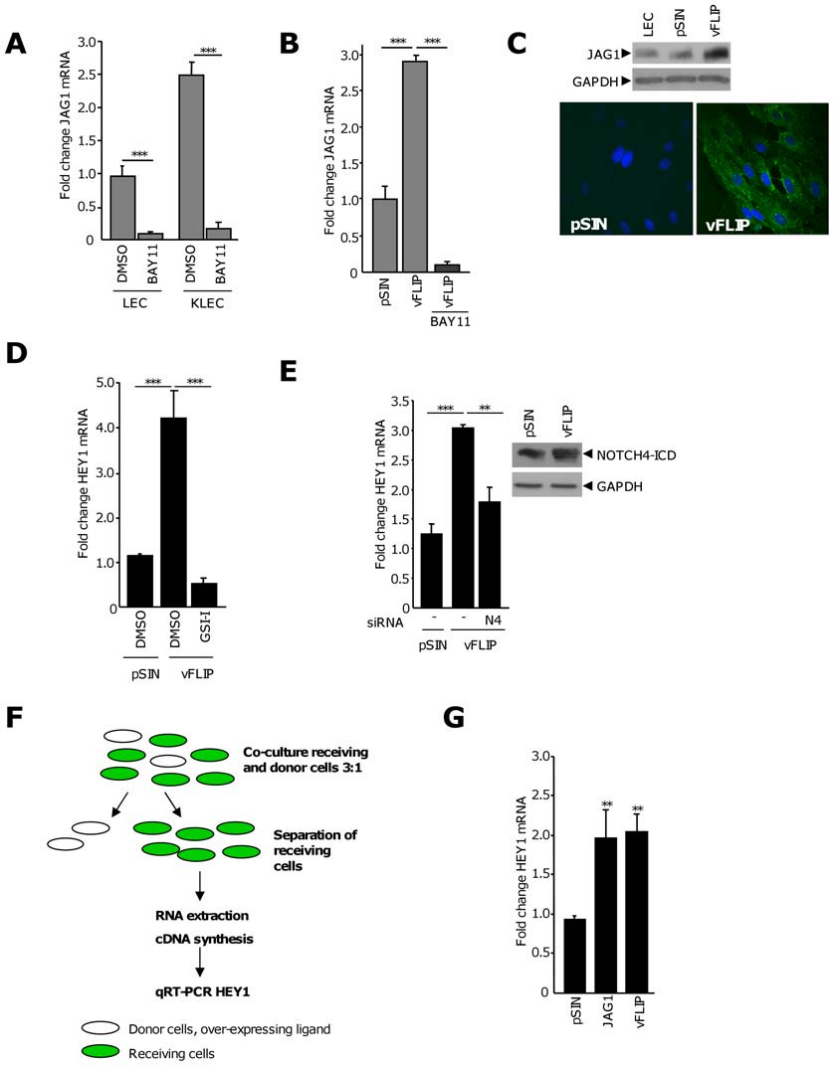
vFLIP induces JAG1 expression in LEC through the NFκB pathway. (A) JAG1 mRNA levels in LEC and KLEC treated with BAY11-7082 (BAY11) or the equivalent volume of DMSO. Columns are the average fold change from two independent experiments. **, *P*<0.01, ***, *P*<0.001, with respect to the corresponding DMSO control. (B) Mean fold change in JAG1 mRNA in pSIN- or vFLIP-expressing LEC treated with BAY11-7082 (BAY11) or the equivalent volume of DMSO. Columns represent the mean fold change from at least 3 independent experiments. ***, *P*<0.01 with respect to DMSO-treated pSIN-infected or vFLIP-infected LEC, indicated by the horizontal bars. (C) Representative western blot and immunoflourescence image showing increased JAG1 protein in pSIN- or vFLIP-LEC. Green, JAG1, blue, DAPI. (D) Mean fold change in HEY1 mRNA in vFLIP-LEC in the presence of γ-secretase inhibitor (GSI-I) or the equivalent volume of DMSO. Columns are the mean fold change from two independent experiments. ***, *P*<0.001, with respect to DMSO-treated pSIN- or vFLIP-infected LEC, indicated by the horizontal bars. (E) Left panel, HEY1 mRNA in LEC transfected with non-silencing siRNA or siRNA targeting NOTCH4 (N4). Columns are the average fold change from three independent experiments. **, *P*<0.01, ***, *P*<0.001, with respect to pSIN or vFLIP-LEC transfected with a non-silencing control, indicated by the horizontal bars. Right panel, levels of NOTCH4-ICD protein in pSIN- and vFLIP-LEC. (F) Schematic representation of the co-culture assay. Ligand-expressing donor cells were generated by infection with the appropriate lentivirus and mixed with receiving cells, stained green, as described according to Materials and Methods. (G) HEY1 mRNA levels in receiving cells co-cultured with LEC over-expressing JAG1 or infected with vFLIP. Columns are the average fold change from three independent experiments. **, *P*<0.01, with respect to cells co-cultured with pSIN-infected LEC.

JAG1 is an NFκB-responsive gene [32] and is induced in endothelial cells through an NFκB-dependent mechanism [8],[11]. Treatment of LEC and KLEC with a chemical inhibitor of the NFκB pathway, BAY11-7082 [33], significantly reduced the basal and KSHV-induced expression of JAG1 in LEC (Figure 2A). These data suggest that the NFκB pathway is important in the maintenance and induction of JAG1 levels. JAG1 expression is not induced in LEC following exposure to KLEC-conditioned media (Figure S2A), suggesting that the KSHV-induced increase in JAG1 in LEC does not occur via a paracrine mechanism.

Increased expression of JAG1 has been suggested to correlate with later-stage KS, specifically plaque and nodular lesions where latent KSHV infection predominates [25],[34]. The restricted number of viral genes expressed during latency includes vFLIP, a potent activator of the NFκB pathway [35]. To examine whether vFLIP can induce JAG1 expression, LEC were infected with lentivirus expressing vFLIP. Increased expression of MHC-I in vFLIP-LEC was used as a control for vFLIP functional expression ([29], data not shown). Compared to control cells infected with pSIN lentivirus, levels of JAG1 mRNA were increased by approximately three-fold in vFLIP-expressing LEC; this induction was abrogated by BAY11-7082 (Figure 2B). vFLIP increased JAG1 protein expression in LEC as measured by western blotting and increased JAG1 was also observed in the spindle-shaped cells characteristic of vFLIP infection (Figure 2C). LEC infected with lentivirus expressing the lytic viral gene K15-P, another inducer of the NFκB pathway [36] did not induce JAG1 expression in LEC (data not shown). Collectively, these data suggest that JAG1 expression is induced primarily by vFLIP through an NFκB-dependent mechanism.

We next investigated whether vFLIP could induce Notch signaling in LEC by measuring levels of the Notch target, HEY1. Expression of vFLIP induced a four-fold increase in HEY mRNA that was abrogated in cells treated with a γ-secretase inhibitor (GSI-I) (Figure 2D), suggesting this induction of HEY1 may depend on canonical Notch signaling. The NOTCH4 receptor expression appeared to be most significant in the context of primary KSHV infection of LEC (Figure 1C). We therefore investigated if the increase in HEY1 by vFLIP was dependent on NOTCH4. vFLIP-expressing LEC had elevated levels of NOTCH4-ICD, detectable by western blot (Figure 2E), and siRNA knockdown of NOTCH4 (Figure S2B) reduced HEY1 mRNA to near basal levels (Figure 2E). These data suggest that vFLIP induces HEY1 expression by way of NOTCH4. Using the co-culture assay described in Figure 2F, we investigated if vFLIP could induce Notch signaling between adjacent cells. HEY1 expression in the receiving cells was assessed by qRT-PCR and was found to be significantly increased in cells exposed to vFLIP or JAG1 donors (Figure 2G). No significant change in another Notch target gene, HES1, was observed. These data suggest that vFLIP can induce HEY1 expression in adjacent cells, through a mechanism involving JAG1 and NOTCH4.

### vGPCR increases levels of DLL4 through an ERK-dependent mechanism

**Figure 3:**
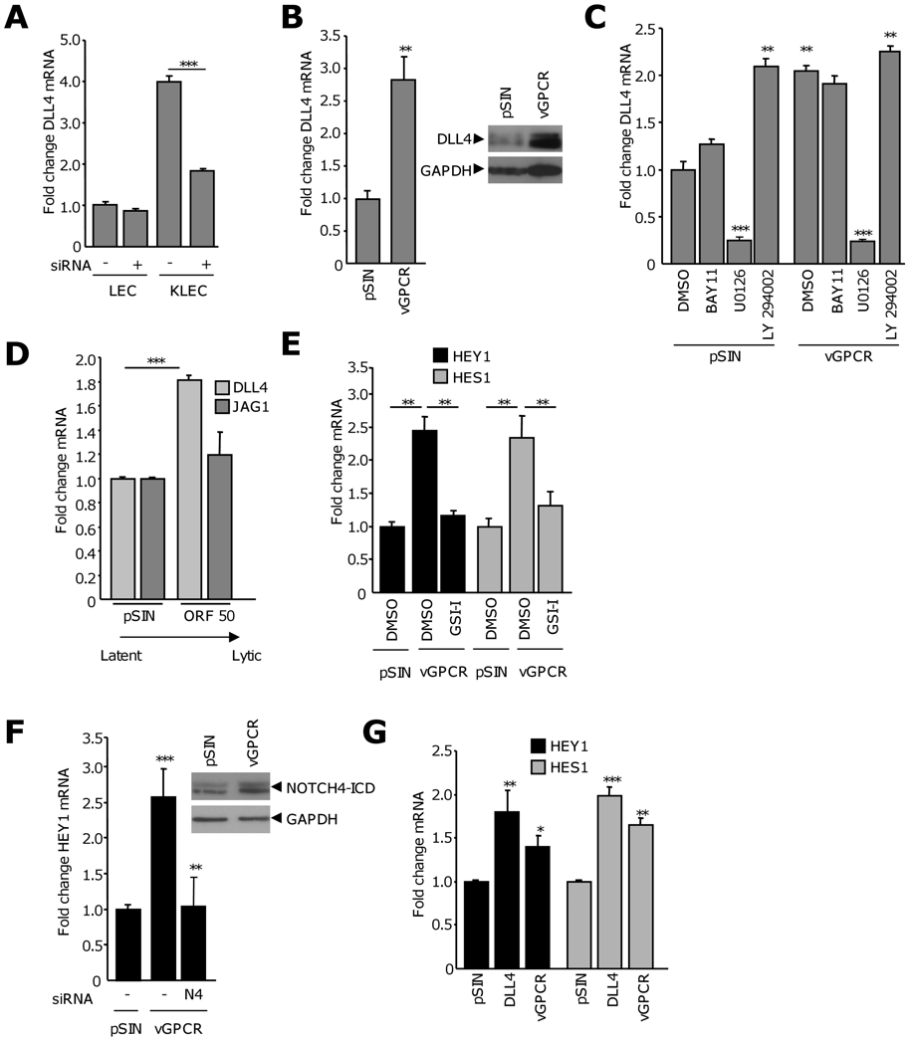
vGPCR induces DLL4 expression in LEC through an ERK-dependent mechanism. (A) DLL4 mRNA levels in LEC and KLEC in the presence of non-silencing (−) or ERK1/2 (+) siRNA. Columns are the average fold change from two independent experiments. ***, *P*<0.001, with respect to the corresponding non-silencing control. *P* = 0.056 between LEC samples. (B) (Left panel) DLL4 mRNA in pSIN- or vGPCR-LEC. Columns are the average fold change from at least three independent experiments. **, *P*<0.01 with respect to pSIN-infected LEC. (Right panel) DLL4 protein in cells infected with pSIN- or vGPCR-expressing lentivirus. (C) DLL4 mRNA levels in pSIN- or vGPCR-LEC treated with the inhibitors BAY11-7082 (BAY11), UO126, LY294002 or the equivalent volume of DMSO. Columns are the average fold change from two independent experiments. ***, *P*<0.001, **, *P*<0.01 with respect to the corresponding DMSO-treated control. (D) DLL4 (light grey bars) and JAG1 (dark grey bars) mRNA levels in BCBL1 cells expressing pSIN or ORF50. Columns, average fold change from three independent experiments. ***, *P*<0.001, with respect to pSIN-expressing control. (E) Mean fold change in HEY1 (black bars) and HES1 (grey bars) mRNA in vGPCR-LEC treated with 5 µM GSI-I or the equivalent volume of DMSO. Columns are the mean fold change from two independent experiments. **, *P*<0.01, with respect to DMSO-treated pSIN- or vGPCR-LEC, indicated by the horizontal bars. (F) (Left panel) HEY1 mRNA levels in vGPCR-infected LEC, transfected with non-silencing siRNA or siRNA targeting NOTCH4 (N4). Columns, average fold change from three independent experiments. **, *P*<0.01, ***, *P*<0.001, with respect to pSIN- or vGPCR-LEC transfected with a non-silencing control, as indicated by horizontal bars. (Right panel) western blot showing elevated levels of NOTCH4-ICD. (G) Expression of HEY1 (black bars) and HES1 (grey bars) mRNA in adjacent cells co-cultured with LEC over-expressing DLL4 or infected with vGPCR. Columns are the average fold change from three independent experiments. *, *P*<0.05, **, *P*<0.01 and ***, *P*<0.001, with respect to cells co-cultured with pSIN-LEC.

DLL4 expression in LEC is not significantly affected by vFLIP (Figure S3A) suggesting an alternative mechanism for its induction in KLEC. The Notch pathway is required for arterial specification [37]–[39], and DLL4 expression is essential for arterial patterning and lymphatic sprouting [40]–[42]. Activation of extracellular-signal regulated kinase (ERK) is also required for the arterial commitment of angioblasts [43],[44] suggesting a functional link between these two pathways. siRNA knock-down of ERK1 and ERK2 (Figure S3B) significantly reduced KSHV-induced DLL4 expression in LEC (Figure 3A) suggesting a role for this pathway in the upregulation of DLL4 during viral infection. The KSHV vGPCR is a potent activator of ERK signaling [45],[46]; levels of DLL4 were therefore investigated in LEC infected with lentivirus expressing vGPCR. Compared to cells expressing pSIN, levels of DLL4 mRNA were increased approximately three-fold in vGPCR-LEC. This increase was also observed at the protein level (Figure 3B).

vGPCR activates multiple signaling cascades [47]. To confirm that the induction of DLL4 by vGPCR is dependent on the ERK pathway, pSIN- and vGPCR-expressing LEC were treated with pharmacological inhibitors of the NFκB, ERK and PI3K pathways (Figure 3C). DLL4 expression was significantly reduced following ERK pathway inhibition only and was unaffected by the BAY11-7082 compound. This agrees with our observation that vFLIP fails to induce DLL4 expression (Figure S3A). Inhibition of the PI3K pathway did not affect the vGPCR-induced increase in DLL4; however, increased basal DLL4 expression was observed (Figure 3C). This may reflect an antagonistic role for PI3K signaling in DLL4 levels in LEC, as has been observed during blood vessel specification [43],[44]. Other KSHV genes can also activate ERK signaling. To investigate the specificity of DLL4 induction by vGPCR, LEC were infected with lentivirus expressing K15-P or Kaposin A as examples of genes known to activate ERK signaling during lytic and latent infection respectively [36],[48]. Neither of these viral genes increased DLL4 levels (Figure S3C). Collectively, these data suggest that vGPCR induces DLL4 expression through an ERK pathway-dependent mechanism.

VEGF can induce DLL4 expression in blood vessel endothelial cells during physiological and pathological angiogenesis [49]–[53] and vGPCR can stimulate VEGF production [54],[55]. We observed a two-fold increase in the expression of VEGF in vGPCR-expressing LEC (Figure S3D); however, LEC grown in vGPCR-conditioned media in the presence or absence of a VEGFR inhibitor, did not show increased DLL4 expression (Figure S3E). LEC did not demonstrate increased levels of DLL4 in response to VEGF at concentrations previously reported to induce DLL4 expression [49],[50],[53] (Figure S3F). These data suggest that the induction of DLL4 in LEC by vGPCR does not occur through a paracrine mechanism involving VEGF and that direct activation of ERK by vGPCR is sufficient to elevate levels of DLL4 in these cells.

JAG1 expression can also be affected by ERK signaling [56], so we investigated levels of JAG1 in vGPCR LEC. We observed reduced levels of JAG1 in these cells (Figure S3G) suggesting that vGPCR preferentially induces DLL4 and this may indicate a role for DLL4 during KSHV lytic infection. To begin to investigate this hypothesis, we expressed KSHV ORF50 in the BCBL1 PEL cell line, which is sufficient to reactivate KSHV from latency [57]. The induction of lytic infection was confirmed by measuring significantly increased expression of ORF50 and the late-lytic gene ORF26. An accompanying 2.5-fold increase in vGPCR expression was also observed (Figure S3H). Compared to control, ORF50 cells expressed significantly more DLL4 (1.8-fold, Figure 3D), while levels of JAG1 remained unchanged. The outcome of Notch signaling, including signaling strength, can be influenced by the type of ligand expressed [58],[59]. These data suggest that DLL4 expression may have a role during the lytic phase of the KSHV cycle and complements signaling established by JAG1 during latency.

DLL4 has been shown to elevate levels of both HES1 and HEY1 in HUVEC [53],[60], so we examined levels of these Notch targets in vGPCR-LEC. Levels of HEY1 and HES1 mRNA were increased about 2.5-fold in vGPCR-LEC and these increases were abrogated following GSI-I treatment (Figure 3E). These data suggest the Notch pathway is involved in the upregulation of HES1 and HEY1 in response to vGPCR. siRNA-mediated silencing of NOTCH4 (Figure S3I) significantly reduced HEY1 expression (Figure 3F), suggesting that vGPCR induces HEY1 through a canonical Notch signaling mechanism involving NOTCH4 and emphasising the importance of this receptor during KSHV infection. In agreement, vGPCR-expressing cells have elevated levels of NOTCH4-ICD protein indicating activation of this receptor (Figure 3F). HES1 expression was not significantly reduced in the presence of NOTCH4 siRNA alone (data not shown) but combined knock-down of NOTCH1 and NOTCH4 significantly reduced HES1 levels and increased levels of NOTCH1-ICD protein were observed in vGPCR-LEC (Figure S3J). These suggest that induction of HES1 in vGPCR-expressing LEC can occur through either NOTCH1 or NOTCH4, indicating a specific role for NOTCH1 in DLL4-stimulated signaling. Utilising our co-culture assay, we examined levels of HES1 and HEY1 in cells adjacent to vGPCR or DLL4-expressing donors. Under both these conditions, HES1 and HEY1 were significantly increased (Figure 3G). These data indicate that DLL4-stimulated Notch signaling can induce HES1 and HEY1 in adjacent LEC and that this signaling is mimicked by vGPCR.

### Notch signaling suppresses the expression of cell cycle components in LEC

**Figure 4:**
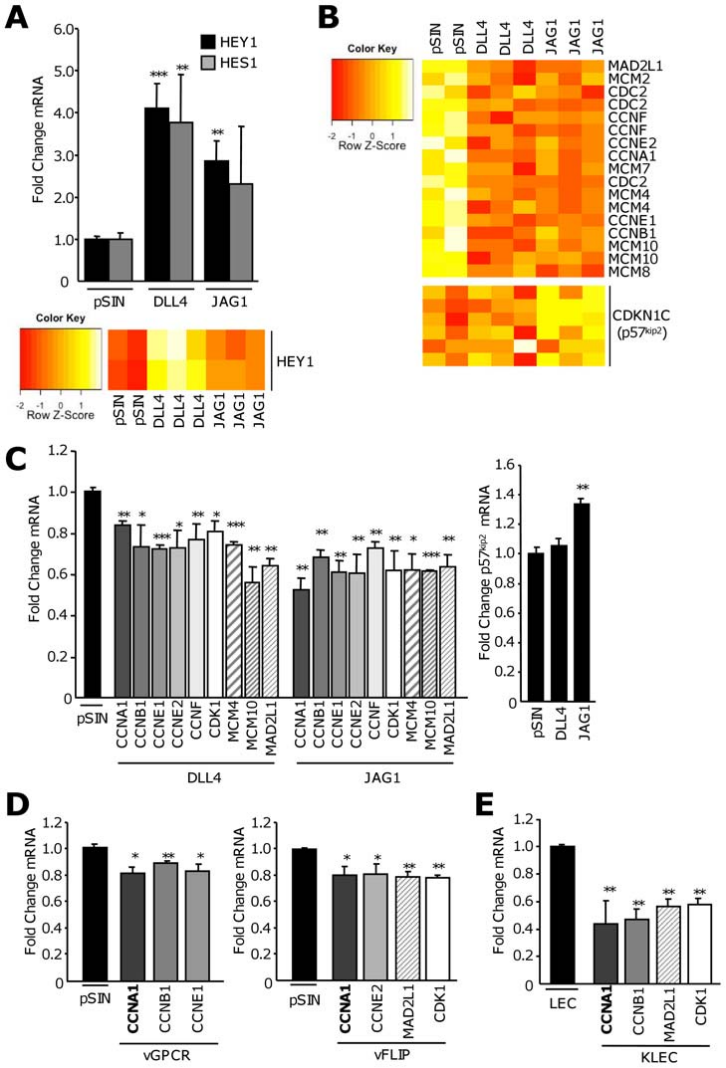
Notch signaling down-regulates cell cycle components in adjacent LEC. (A) (Top panel) Mean fold change in HEY1 (black bars) and HES1 (grey bars) mRNA in receiving LEC co-cultured with either DLL4- or JAG1-overexpressing donor cells. Columns are the mean fold change from three independent co-culture experiments. cDNA from these experiments was hybridised to HG-U133 Plus 2.0 Array (Affymetrix) ***, *P*<0.001, and **, *P*<0.01, with respect to receiving cells co-cultured with pSIN-LEC. (Bottom panel) Heatmap based on gene expression array profiling representing HEY1 expression in receiving cells co-cultured with pSIN, DLL4 or JAG1 in triplicate. Rows correspond to two HEY1 probe sets; red and yellow denote low and high expression respectively, *P*<0.01. (B) (Top panel) Heatmap illustrating the relative changes in expression of cell cycle genes significantly altered in LEC co-cultured with DLL4 and JAG1 compared to pSIN. (Bottom panel) Heatmap illustrating the specific upregulation of CDKN1C (p57^Kip2^) in JAG1-stimulated LEC according to six probe sets *P*<0.01. (C) (Top panel) mRNA levels of cell cycle components altered by DLL4 and JAG1-induced Notch signaling in receiving LEC. Columns are the mean fold change from three independent co-culture experiments, values are normalised to LEC co-cultured with pSIN. ***, *P*<0.001, **, *P*<0.01 and *, *P*<0.05 with respect to pSIN co-culture. CCN, cyclin; MCM, minichromosome maintenance protein; CDK1, cyclin dependent kinase 1. (Bottom panel) Mean fold change of p57^Kip2^ mRNA in LEC stimulated by DLL4 or JAG1 compared to pSIN. **, *P*<0.01 with respect to pSIN co-culture. (D) Expression of cell cycle genes in LEC co-cultured with vGPCR- (left panel) or vFLIP-expressing cells. Columns are the average of at least two independent experiments. **, *P*<0.01 and *, *P*<0.05 with respect to pSIN. (E) mRNA levels of cell cycle genes suppressed in LEC following KSHV infection. Columns are the average of two independent experiments with respect to LEC. **, *P*<0.01 and *, *P*<0.05.

To investigate the role of DLL4- and JAG1-stimulated Notch signaling in LEC, we performed gene expression microarray (GEM) analysis on LEC co-cultured with DLL4- or JAG1-expressing donors. The ligands were expressed to equivalent levels in donor cells as analysed by western blot (Figure S4A). Stringent selection (false discovery rate threshold q<0.05) generated a list of the most significantly altered genes in the receiving cells as a consequence of exposure to DLL4 or JAG1 (Table S1). We confirmed the GEM data by validating members of this genelist by qRT-PCR and observed increased expression of CD38 and LYVE1, and decreased levels of NRP1, a known target of DLL4-induced Notch signaling [60],[61] (Figure S4B). We confirmed that receiving cells upregulated Notch target genes in response to both DLL4 and JAG1 compared to pSIN (Figure 4A). In agreement with our previous data, DLL4 stimulated significant increase in HEY1 and HES1 expression (approximately four-fold) whereas JAG1 resulted in significant increase in HEY1 only (nearly three-fold). These data indicate that DLL4 induced a more pronounced change in Notch target gene expression, which is reflected in the heatmaps from two HEY1 probes (Figure 4A). Similarly, when the 165 genes most significantly altered in response to DLL4 are considered, (Figure S4C and Table S2); these changes are mimicked in JAG1-stimulated cells but are less pronounced.

Less stringent selection using an unadjusted *P* threshold of 0.005 generated a larger data set of significantly regulated genes on which gene ontology analysis was performed using GENECODIS [62]. This analysis indicated that, amongst genes suppressed in cells stimulated by both DLL4 and JAG1, cell cycle, cell division and mitotic pathways were enriched. Specifically, cyclins (CCN), cyclin-dependent kinase 1 (CDK1), mitotic arrest deficient-like 1 (MAD2L1) and MCM proteins, were indicated by the GEM data as significantly down-regulated in both JAG1- and DLL4-receiving cells (Figure 4B). The CKI p57^Kip2^ (CDKN1C) was uniquely upregulated by JAG1 (Figure 4B); no significant change in other CKIs, such as p21^Cip1^, p27^Kip1^ or members of the INK4 family, was observed, despite associations with Notch signaling in other systems [16],[63]. Significant down-regulation of CCNA1, CCNB1, CCNE1, CCNE2 and CCNF; CDC2 (CDK1), MCM4, MCM10 and MAD2L1 was confirmed by qRT-PCR for both JAG1- and DLL4-receiving cells (Figure 4C, left panel). The JAG1-dependent increase in p57 predicted by GEM analysis was also confirmed (Figure 4C, right panel). MAD2L1 is part of a six-gene expression signature, including the upregulation of HES1, characteristic of quiescence triggered by a variety of arrest signals [64]. The co-ordinated expression of p57^Kip2^ and HES1 has also been associated with quiescence reversibility [14],[15]. Collectively, these observations suggest a role for DLL4 and JAG1 in manipulating the cell cycle in adjacent LEC.

To investigate whether the changes observed in Notch ligand-stimulated cells were recapitulated by vGPCR or vFLIP, we measured the expression of these genes in receiving cells co-cultured with vGPCR- or vFLIP-expressing LEC (Figure 4D). We confirmed significant reduction in CCNA1, CCNB1, CCNE1, CCNE2, MAD2L1 and CDK1 in response to vGPCR and vFLIP co-culture respectively. CCNA1 expression was reduced in all four co-culture conditions. To investigate a role for the suppression of these genes in the context of KSHV infection, we measured their expression in KLEC and observed that these genes were significantly down-regulated (Figure 4E), with the exception of the E-type cyclins (not shown). Significantly reduced expression of CCNF, MCM4 and MCM10 was also observed in KLEC (Figure S4D). These data suggest that cell cycle components are targets of Notch signaling in LEC and are suppressed in cells adjacent to those expressing KSHV viral genes, suggesting that Notch signaling may influence the cell cycle in cells adjacent to those infected by KSHV.

## Discussion

The expression of Notch signaling components has been reported in KS, but the molecular mechanisms underlying the activation of this pathway by KSHV have not been established. Here we show that KSHV manipulates canonical Notch signaling in LEC by increasing the expression of JAG1 and DLL4 through vFLIP and vGPCR. The vFLIP-induced increase in JAG1 occurs through an NFκB-dependent mechanism and mimics the induction of blood vessel tip cells during pathological angiogenesis by TNF [8],[11]. This provides a new example of the manipulation of a host endothelial signaling mechanism by KSHV. vGPCR is a multifunctional protein, but here we show that its induction of DLL4 is specifically ERK-dependent. How ERK signaling relates to the Notch pathway in endothelial cells has previously been unclear and our data indicate a direct link between ERK and DLL4 expression in LEC. Interestingly, our data also indicate that the induction of DLL4 in LEC is unlikely to occur as a result of VEGF stimulation. This is also the first report of a functional association between KSHV and DLL4.

We show that the increase in levels of the Notch target gene, HEY1, in KLEC occurs through NOTCH4. Whereas there is an established functional association between DLL4, NOTCH4 and NOTCH1 in terms of expression patterns [9],[40],[49],[65], JAG1 has been shown to be a ligand for multiple Notch receptors, but not directly for NOTCH4 [66],[67]. The induction of HEY1 in response to vFLIP is dependent on NOTCH4, confirming an association between JAG1 and NOTCH4 in LEC. We show that DLL4 can induce expression of an additional Notch target gene, HES1, though a mechanism dependent on NOTCH1 and NOTCH4, indicating a specific role for NOTCH1 in LEC.

The outcome of Notch signaling, including signaling strength, can be influenced by the type of ligand expressed [59]. Using gene expression profiling, we show that the most significant changes in gene expression elicited by DLL4 in adjacent cells are more pronounced compared to the changes elicited in the same genes by JAG1. HEY1 and HES1 are basic helix-loop-helix transcription factors that can heterodimerise to enhance Notch signaling effects [68]–[70]. The induction of both HES1 and HEY1 by DLL4 could explain why signaling induced by this ligand is more potent than JAG1.

Our data also indicate a distinct role for these Notch ligands during latent (vFLIP) and lytic (vGPCR) infection of LEC by KSHV. The majority of cells in KS are latently infected with virus, whereas lytic infection is short-lived and only accounts for a small percentage of cells [23]. In addition, vGPCR-induced transcripts are associated with limited temporal expression [71]. The periodic expression of DLL4 during lytic infection may contribute to “topping up” Notch signaling established by JAG1 during latency. The increased potency of DLL4-induced signaling may compensate for its potentially restricted expression to permit functional signaling. The functional outcome of DLL4-stimulated signaling is dose-dependent [40],[65],[72],[73] and can operate through distinct spatial expression patterns. [13],[58]. While complementary roles for DLL4 and JAG1 have been suggested during angiogenesis [58], a mechanism through which expression of these ligands can be differentially regulated in this context has not been determined. Our work suggests that KSHV can establish differential upstream signaling events leading to the expression of DLL4 and JAG1 coincident with lytic and latent infection respectively.

Gene ontology analysis of our expression profiling data did not indicate significant overall changes in angiogenesis-associated genes in either DLL4- or JAG1-stimulated cells. However, both ligands elicited significant suppression of the expression of cell cycle components in adjacent LEC. A number of these genes were also suppressed in LEC adjacent to vFLIP- and vGPCR-LEC and were down regulated in KLEC. Cyclin A1 (CCNA1) expression was suppressed under all these conditions and has been indicated as a target of activation of NOTCH1 [74]. Cyclin A1 is functionally associated with multiple cell cycle components including CDK1 [75]–[77], which is also suppressed in our system. Suppression of cyclin A induces cell cycle arrest in arterial endothelial cells [78].

**Figure 5:**
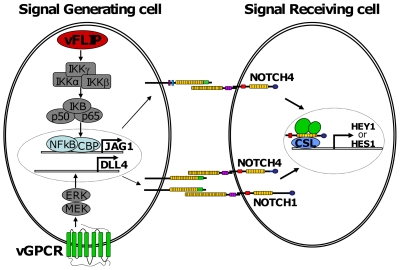
Proposed model describing induction of Notch signaling by KSHV latent (vFLIP) and lytic (vGPCR) infection. Latent expression of vFLIP and lytic expression of vGPCR induce JAG1 and DLL4 expression in infected cells so that they become Notch signal generating cells. Interaction between JAG1 and NOTCH4 and DLL4 and NOTCH4 or NOTCH1 stimulates adjacent, uninfected cells to become signal receiving cells, in which Notch target genes are upregulated.

The effect of the Notch pathway on the cell cycle has been associated with quiescence and reduced proliferation in a number of systems [15]–[19],[61],[63],[79],[80]. In the context of KS, suppression of cell cycle components could provide a growth advantage to infected (signal generating) cells over uninfected surrounding cells (Figure 5). Alternatively, instigation of Notch signaling in adjacent immune cells could halt them to provide a means of immune escape for the virus. To fully address the effect of KSHV-induced Notch signaling on modulation of the host immune response to KS would require an immunocompetent model of KS, which does not yet exist.

Our data also show that JAG1-induced Notch signaling increases p57^Kip2^ expression in adjacent LEC. Inspection of the p57^Kip2^ promoter reveals two CSL binding sites (data not shown), making this gene a potentially direct target for JAG1-induced activation of Notch in LEC. The co-ordinated expression of p57^Kip2^ and HES1 has been associated with quiescence reversibility [14],[15]. Our predicted periodic expression of DLL4 provides the potential for co-ordination between p57^Kip2^ and HES1 in KS, suggesting a mechanism by which KSHV may influence the plasticity of the surrounding cells during lytic infection, thereby making them more susceptible to reprogramming by the virus [14],[15],[20],[81].

Our co-culture model is representative of the “tip hypothesis” of branching angiogenesis, whereby the tip cells of developing vessels express ligand and signal to adjacent cells to adopt the quiescence-associated stalk phenotype [50], [82]–[84]. The presence of specialised tip cells has not been described for developing lymphatic vessels [85], but DLL4 has been implicated in lymphatic sprouting [42]. Our findings indicate a potential role for DLL4 and JAG1 in sprouting lymphangiogenesis. Furthermore, elucidation of the mechanism by which canonical Notch signaling is manipulated by KSHV in LEC raises the possibility that KS may be susceptible to treatment with the NOTCH1 Decoy [86], an inhibitor of NOTCH1 and NOTCH4 signaling, shown to be effective in neuroblastoma and mouse mammary carcinoma xenografts. Anti-DLL4 antibodies have been reported to reduce tumour size in multiple tumour xenograft models [51],[87] and could also be therapeutically relevant in KS treatment. Further studies modelling KSHV-induced oncogenesis in the context of KSHV deletion mutants [88],[89] or in the presence of these Notch pathway inhibitors or would provide insight into this pathway as a possible target in the management of KS.

## Materials and Methods

### KSHV production and infection of LEC

LEC were cultured as described and KSHV was produced from BCBL1 cells and used to infect LEC as previously described ([29],[30] and Protocol S1). GFP expression in KLEC was used as an indicator of KSHV infection; GFP-positive KLEC was typically approximately 35%, three days post-infection (p.i.).

### Lentiviral expression of KSHV genes, DLL4 and JAG1

KSHV genes were cloned from the BC3 and BC1 PEL cell lines and were expressed using a modified pSIN-MCS lentiviral vector and produced in 293T cells as previously described [29],[30]. DLL4 and JAG1 cDNAs were cloned from HUVEC cDNA into pSIN-MCS as described in Protocol S1. Lentiviral copies per cell were determined by qPCR and a maximum of ten copies per cell were used to avoid cytopathic effects. All experiments shown were performed three days post-lentiviral infection (p.i.).

### Co-culture assays and cell sorting

LEC were infected with the appropriate lentivirus as described to generate the ligand-expressing (signal generating) cells. LEC to be designated “receiving cells” were stained with CellTracker Green CMFDA (Invitrogen) diluted to a final concentration of 5 µM in Optimem (Invitrogen). Receiving cells were mixed with ligand-expressing donor cells on 10 cm dishes at a ratio of 3∶1 [90],[91] and co-cultured for 60 hours. Pure populations of signal-receiving cells were obtained by flow cytometric sorting of the CellTracker-labelled LEC (MoFlo XDP, Beckman-Coulter) directly into Qiazol Lysis Reagent (Qiagen). RNA extraction was performed as described [30].

For GEM experiments, one10 cm dish was used per Affymetrix chip and 1 µg of total RNA was used to generate cDNA using T7-linked oligo(dT) primer and the custom SuperScript dscDNA synthesis kit (Invitrogen). After second-strand synthesis, *in vitro* transcription was carried with biotinylated UTP and CTP using GeneChip® IVT Labeling Kit (http://www.affymetrix.com/support/technical/technotes/ivt_technote.pdf)

### RNA interference

LEC were seeded in 5×10^4^ cells per well in six-well plates one day prior to transfection with 100 nM NOTCH1- or NOTCH4-tagetting or non-targeting siRNA (OnTargetPlus SmartPool, Dharmacon). Transfections were performed using Oigofectamine reagent (Invitrogen). Cells were infected with KSHV 48 hours post siRNA transfection or with the appropriate lentivirus 24 hours post-transfection.

### Pharmacologic inhibitors

The following chemical inhibitors were used: BAY11-7082 (NFκB pathway inhibitor, 5 µM), JNK inhibitor II (25 µM), SB202190 (p38/MAPK inhibitor, 10 µM), UO126 (MEK inhibitor, 10 µM), LY294002 (PI3K inhibitor, 5 µM) and γ-secretase Inhibitor I (GSI-I, Z-Leu-Leu-Nle-CHO (Nle = Norleucine), 5 µM), all from Calbiochem. For LEC and KLEC at 72 hours p.i., and LEC infected with lentivirus, the inhibitors were added to the cells for 6 hours, apart from BAY11-7082 and LY294002, which were added for 2 hours and 4 hours, respectively.

### qRT-PCR and qPCR

Extraction of genomic DNA and RNA was performed using QIAamp DNA mini and RNEasy mini kits (Qiagen) respectively. DLL4, JAG1, NOTCH4, HEY1 and HES1 mRNA levels were quantified by qRT-PCR using Taqman Gene Expression Assays (Applied Biosystems). GAPDH was used as a housekeeping reference gene and quantified using the SYBR Green Master Mix (Applied Biosystems) and optimised forward and reverse primers at a final concentration of 300 nM. Levels of genes highlighted in the co-culture microarray were quantified in the same way as GAPDH (primers listed in Table S2). qPCR for lentiviral copy number was performed as described [30].

### Western blotting and immunofluorescence microscopy

Cells were lysed on ice for 30 minutes in buffer (PBS containing 1% NP40 and 0.1% SDS, supplemented with Protease Inhibitor Cocktail (Sigma)) before clearing by centrifugation. Western blotting was performed as described [30] using equal amounts of total protein (20 µg–30 µg) per sample. The following antibodies were used: goat anti-JAG1 (C-20, 1∶500), rabbit anti-NOTCH4 (H225, 1∶200) from Santa Cruz Biotechnology; rabbit anti-DLL4, rabbit anti-cleaved NOTCH1 (both 1∶1,000) from Cell Signalling Technology; mouse anti-GAPDH (6C5, 1∶5,000) from Advanced Immunochemical Inc. Secondary antibodies were from DAKO and used at a dilution of 1∶5,000.

For immunofluorescent assay (IFA), cells were fixed and permeabilised using formalin 3.7% and PBS-T-0.1% Triton X-100, and slides were stained as previously described [92]. The anti-JAG1 antibody was used at 1∶50 and anti-goat-FITC (DAKO) was used at 1∶200. Images were taken using an Ultra*VIEW* ERS confocal microscope (Perkin Elmer).

### GEM analysis

Affymetrix hgu133plus2 GEM data was background corrected, normalised and summarised using the robust multiarray average (rma) algorithm [93], from the Bioconductor ‘*affy*’ package for R [94]. All subsequent analyses and plots show Log2 expression units. Statistical analyses, p-values and false discovery rates where shown were calculated using the ‘*limma*’ package, again from Bioconductor [95]. Where expression values are shown as a heatmap the data has been row scaled with standardised expression values (Z-scores) obtained for each probeset by subtracting the mean of each row and dividing this by the standard deviation. KLEC GEM profiles of six pairs of LEC and KLEC were generated and analyzed as described [29]. KLEC GEM data are available in the ArrayExpress database with accession numbers E-MEXP-561. Co-culture GEM data have been submitted to Gene Expression Omnibus (GEO) and assigned accession number GSE16547.

### Statistics

All experiments were performed in independent replicates and error bars correspond to standard deviation from the mean. Statistical significance (*P* values) was calculated with a two-sided unpaired Student's *t* test. Statistical analysis of the KLEC GEM was performed as described using a moderated *t* statistic and a false discovery rate correction [29].

## Supporting Information

Figure S1 Knock-down of NOTCH (diamond) and NOTCH4 (square) mRNA in KLEC by siRNA targeting NOTCH (N1) or NOTCH4 (N4). mRNA quantified by qRT-PCR with respect to expression in KLEC transfected with non-silencing (−) siRNA. Data points are the average of three independent experiments; bars are the standard deviation from the mean. Significant knock-down of NOTCH and NOTCH4 in the presence of the appropriate siRNA calculated by a two-sided *t* test with respect to non-silencing control, **, p<0.01, ***, p<0.001.
(0.25 MB TIF)

Figure S2 (A) Relative change in JAG1 mRNA in LEC grown in LEC-conditioned medium or KLEC-conditioned medium for 48 hours. Columns are the average fold change from three independent experiments. (B) Expression of NOTCH4 mRNA in vFLIP-infected LEC transfected with nonsilencing (−) siRNA or NOTCH4 (N4)-targeting siRNA. Data points are the average of three independent experiments; bars are the standard deviation from the mean. mRNA levels measured by qRT-PCR with respect to vFLIP-LEC transfected with non-silencing siRNA. Significant knock-down of NOTCH4 calculated by a two-sided *t* test with respect to non-silencing control, *P* = 0.014.
(0.26 MB TIF)

Figure S3 (A) Relative levels of DLL4 mRNA in LEC infected with lentivirus expressing vFLIP or pSIN control. Columns are the average fold change from at least three independent experiments; bars are the standard deviation. (B) Expression of ERK1 (dark grey bars) and ERK2 (light grey bars) in LEC and KLEC transfected with non-silencing (−) or ERK1/2-tagetting (+) siRNA. Columns are the average fold change from two independent experiments. Significant knock-down of ERK1/2 is indicated with respect to the corresponding non-silencing control. *, *P*<0.05, **, *P*<0.01. (C) Relative levels of DLL4 mRNA in LEC infected with lentiviruses expressing KSHV genes (K15 or Kaposin A) or pSIN control. LEC infected with a maximum of 10 lentiviral copies per cell. Columns are the average fold change from at least three independent experiments. (D) Relative levels of VEGF mRNA in pSIN- and vGPCR-expressing LEC. Columns are the average fold change from two independent experiments. **, *P*<0.01. (E) Relative levels of DLL4 in LEC cultured in pSIN- or vGPCR-conditioned media in the presence (+) or absence (−) of a VEGFR inhibitor. Columns are the average fold change from two independent experiments with respect to DMSO-treated pSIN control. (F) DLL4 mRNA levels in LEC treated with increasing concentrations of VEGF_165_ at the indicated time points. Columns are the average fold change from two independent experiments with respect to untreated LEC control. (G) JAG1 mRNA levels in LEC infected with pSIN or vGPCR lentivirus. Columns are the average fold change from three independent experiments; bars are the standard deviation. Significance with respect to pSIN. **, *P*<0.01. (H) mRNA levels of ORF50 (white bars), ORF26 (light grey bars) and vGPCR (dark grey bars) in BCBL1 cells infected with pSIN-expressing (−) or ORF50-expressing (+) lentivirus. Columns are the average fold change from three independent experiments. Significance with respect to pSIN. **, *P*<0.01, ***, *P*<0.001. (I) Level of NOTCH4 mRNA in vGPCR-infected LEC transfected with non-silencing (−) siRNA or NOTCH4 (N4)-targeting siRNA. Data points are the average of three independent experiments; bars are the standard deviation from the mean. mRNA levels measured by qRT-PCR with respect to vGPCR-LEC transfected with nonsilencing siRNA. Significant knock-down of NOTCH4 with respect to non-silencing control, *P* = 0.030. (J) (Left panel) Levels of HES1 mRNA in LEC infected with pSIN- or vGPCRexpressing lentivirus in the presence of non-silencing (−) siRNA control or siRNA targeting NOTCH and NOTCH4 (N1/N4). Columns are the average fold change from three independent experiments. Significance with respect to non-silencing control *, *P*<0.05. (Middle panel) Western blot of NOTCH-ICD in vGPCR-LEC detected using an antibody specific for NOTCH cleaved at Val1744. (Right panel) Knock-down of NOTCH (diamond) and NOTCH4 (square) mRNA in vGPCR-LEC by siRNA targeting NOTCH (N1) or NOTCH4 (N4). mRNA quantified by qRT-PCR with respect to expression in vGPCR-LEC transfected with nonsilencing (−) siRNA. Data points are the average of two independent experiments; bars are the standard deviation from the mean. Significant knock-down of NOTCH and NOTCH4 calculated by a two-sided *t* test with respect to non-silencing control, *, p<0.05, **, p<0.01.
(1.32 MB TIF)

Figure S4 (A) Western blot of protein expression levels of DLL4 and JAG1 in Donor LEC used in the co-culture assay for the microarray. (B) (Left panel) levels of CD38 (dark grey bars) and LYVE1 (light grey bars) mRNA in receiving LEC stimulated by DLL4 or JAG1. (Right panel) levels of NRP1 mRNA in DLL4-stimulated LEC. Gene expression measured by qRT-PCR relative to cells cocultured with pSIN-expressing donor LEC. Columns are the average fold change from three independent co-culture assays; bars are the standard deviation from the mean. **, p<0.01,*, p<0.05 with respect to pSIN co-culture. (C) Heat map of the relative changes in expression of genes most significantly altered in LEC co-cultured with DLL4 compared to pSIN and the corresponding changes in cells receiving JAG1-induced signals. Gene probes are ordered according to the list highlighted in Table S1. (D) mRNA levels of cyclin F (CCNF), MCM10 and MCM4 in KLEC. Columns are the average of two independent experiments with respect to LEC. **, *P*<0.01 and *, *P*<0.05.
(1.13 MB TIF)

Table S1 List of the most significantly altered genes in LEC receiving DLL4 or JAG1 signals relative to pSIN. Stringent selection of genes altered in Receiving LEC using normal p value adjustment (q<0.05).
(0.07 MB XLS)

Table S2 List of the most significantly altered genes in LEC receiving DLL4 signals relative to pSIN. Gene order corresponds to the probes illustrated in Figure S4C. Highlights correspond to genes upregulated (yellow) and down-regulated (pink). M is the difference in mean expression DLL4-pSIN; the *t* and P values were calculated using limma.
(0.04 MB XLS)

Table S3 Primer sequences used for qRT-PCR of the indicated genes and for cloning of DLL4 and JAG1 from HUVEC cDNA into pSIN. For cloning primers, underlined sequence indicates junk DNA; bold sequence indicates the restriction site (BamHI for forward primers, NotI for reverse).
(0.02 MB XLS)

Protocol S1 Supporting protocols.
(0.02 MB PDF)
