# Supplementary figures and images for: KSHV Manipulates Notch Signaling by DLL4 and JAG1 to Alter Cell Cycle Genes in Lymphatic Endothelia
Source: PLoS Pathog. 2009 Oct 9;5(10):e1000616. doi: 10.1371/journal.ppat.1000616 (PMC2751827; doi:10.1371/journal.ppat.1000616)

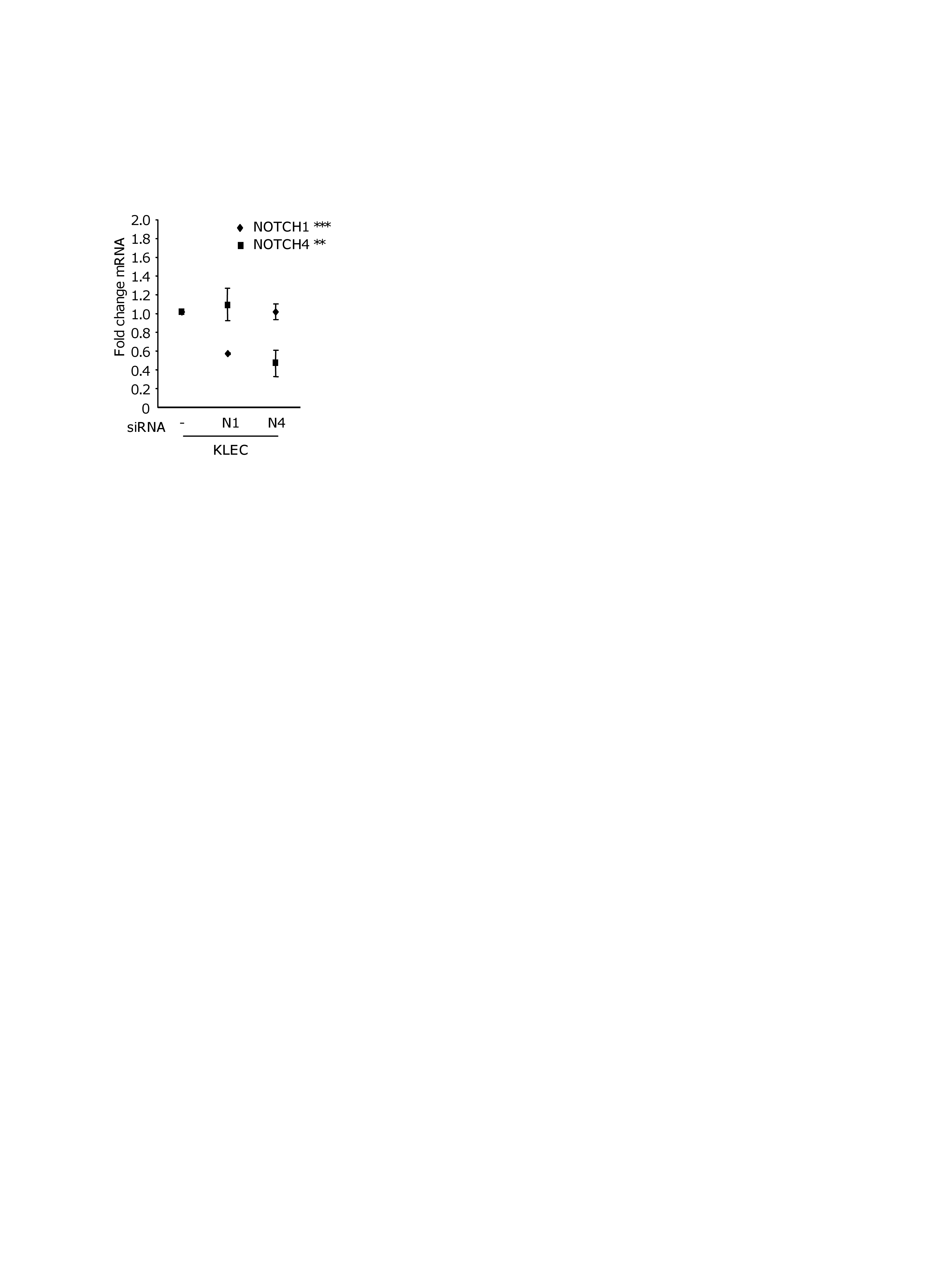

Supplement: Figure S1 — Knock-down of NOTCH (diamond) and NOTCH4 (square) mRNA in KLEC by siRNA targeting NOTCH (N1) or NOTCH4 (N4). mRNA quantified by qRT-PCR with respect to expression in KLEC transfected with non-silencing (−) siRNA. Data points are the average of three independent experiments; bars are the standard deviation from the mean. Significant knock-down of NOTCH and NOTCH4 in the presence of the appropriate siRNA calculated by a two-sided t test with respect to non-silencing control, **, p<0.01, ***, p<0.001. (0.25 MB TIF) [file ppat.1000616.s001.tif]

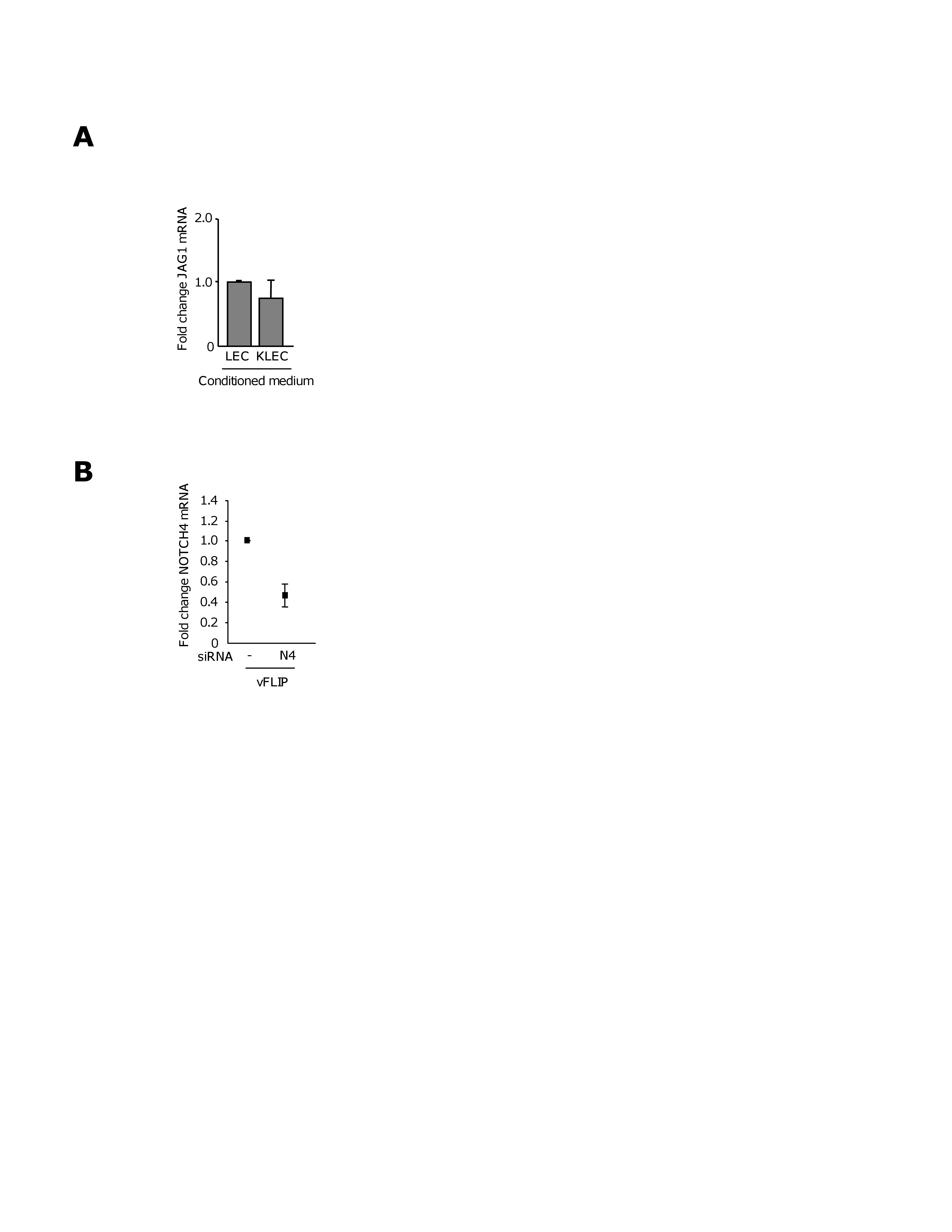

Supplement: Figure S2 — (A) Relative change in JAG1 mRNA in LEC grown in LEC-conditioned medium or KLEC-conditioned medium for 48 hours. Columns are the average fold change from three independent experiments. (B) Expression of NOTCH4 mRNA in vFLIP-infected LEC transfected with nonsilencing (−) siRNA or NOTCH4 (N4)-targeting siRNA. Data points are the average of three independent experiments; bars are the standard deviation from the mean. mRNA levels measured by qRT-PCR with respect to vFLIP-LEC transfected with non-silencing siRNA. Significant knock-down of NOTCH4 calculated by a two-sided t test with respect to non-silencing control, P = 0.014. (0.26 MB TIF) [file ppat.1000616.s002.tif]

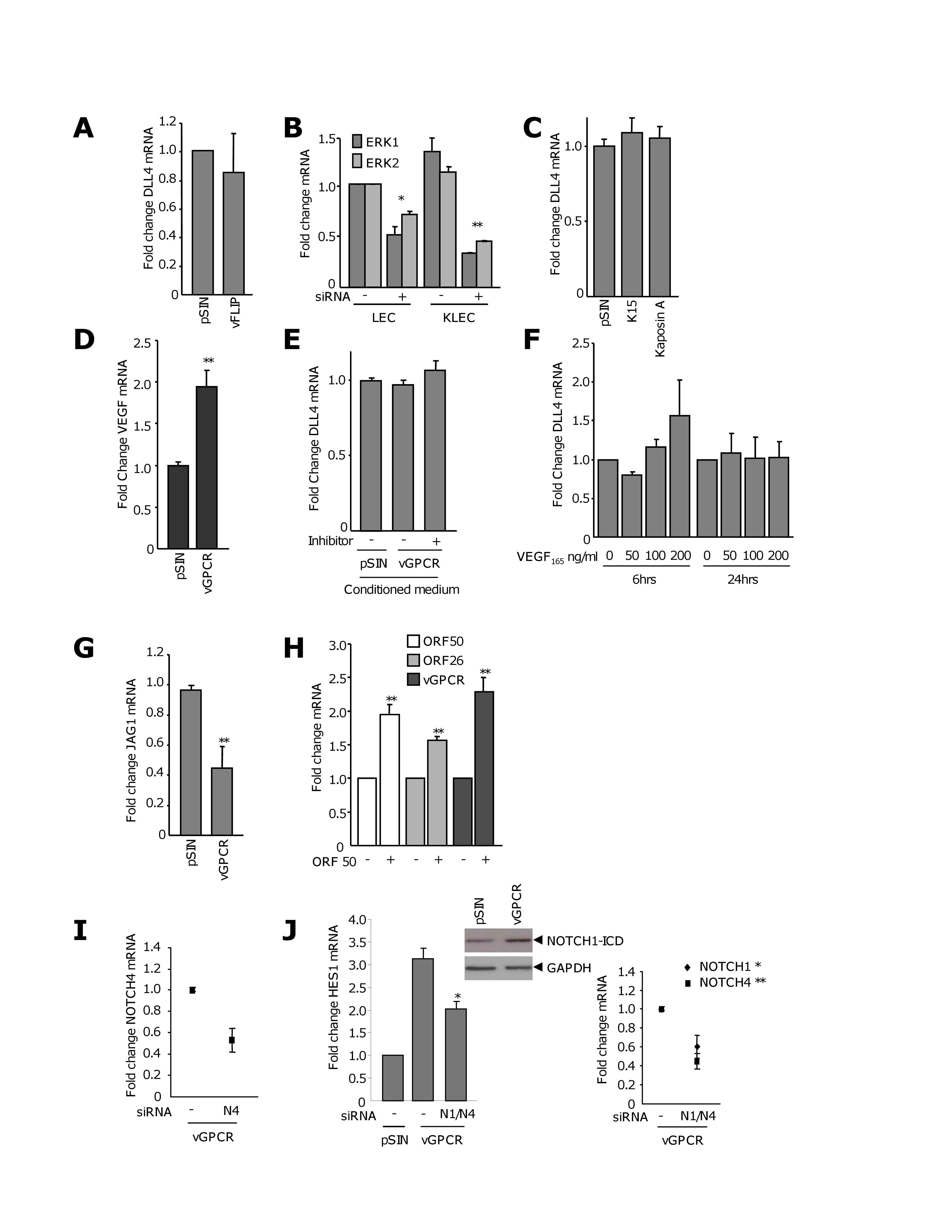

Supplement: Figure S3 — (A) Relative levels of DLL4 mRNA in LEC infected with lentivirus expressing vFLIP or pSIN control. Columns are the average fold change from at least three independent experiments; bars are the standard deviation. (B) Expression of ERK1 (dark grey bars) and ERK2 (light grey bars) in LEC and KLEC transfected with non-silencing (−) or ERK1/2-tagetting (+) siRNA. Columns are the average fold change from two independent experiments. Significant knock-down of ERK1/2 is indicated with respect to the corresponding non-silencing control. *, P<0.05, **, P<0.01. (C) Relative levels of DLL4 mRNA in LEC infected with lentiviruses expressing KSHV genes (K15 or Kaposin A) or pSIN control. LEC infected with a maximum of 10 lentiviral copies per cell. Columns are the average fold change from at least three independent experiments. (D) Relative levels of VEGF mRNA in pSIN- and vGPCR-expressing LEC. Columns are the average fold change from two independent experiments. **, P<0.01. (E) Relative levels of DLL4 in LEC cultured in pSIN- or vGPCR-conditioned media in the presence (+) or absence (−) of a VEGFR inhibitor. Columns are the average fold change from two independent experiments with respect to DMSO-treated pSIN control. (F) DLL4 mRNA levels in LEC treated with increasing concentrations of VEGF165 at the indicated time points. Columns are the average fold change from two independent experiments with respect to untreated LEC control. (G) JAG1 mRNA levels in LEC infected with pSIN or vGPCR lentivirus. Columns are the average fold change from three independent experiments; bars are the standard deviation. Significance with respect to pSIN. **, P<0.01. (H) mRNA levels of ORF50 (white bars), ORF26 (light grey bars) and vGPCR (dark grey bars) in BCBL1 cells infected with pSIN-expressing (−) or ORF50-expressing (+) lentivirus. Columns are the average fold change from three independent experiments. Significance with respect to pSIN. **, P<0.01, ***, P<0.001. (I) Level of NOTCH4 mRNA in vGP [file ppat.1000616.s003.tif]

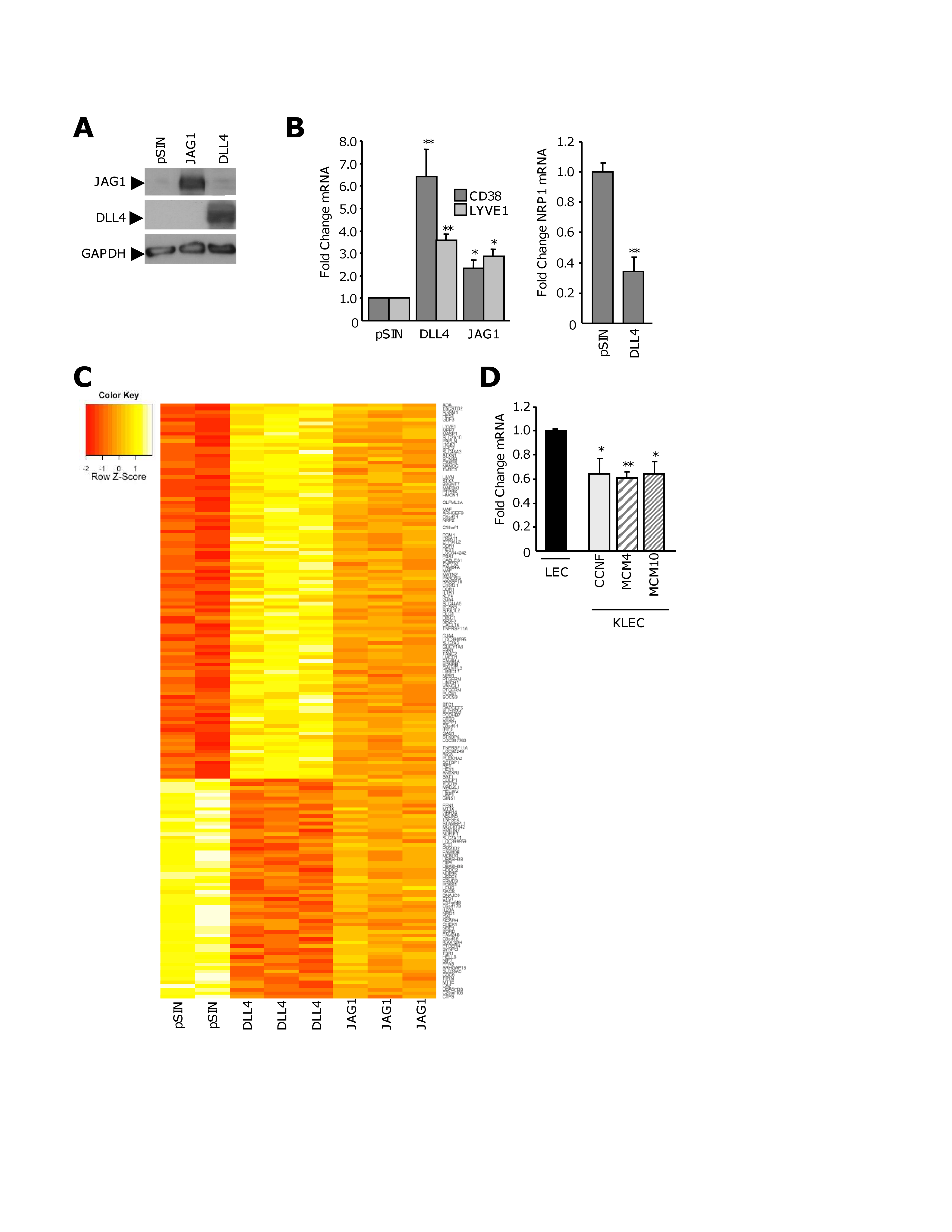

Supplement: Figure S4 — (A) Western blot of protein expression levels of DLL4 and JAG1 in Donor LEC used in the co-culture assay for the microarray. (B) (Left panel) levels of CD38 (dark grey bars) and LYVE1 (light grey bars) mRNA in receiving LEC stimulated by DLL4 or JAG1. (Right panel) levels of NRP1 mRNA in DLL4-stimulated LEC. Gene expression measured by qRT-PCR relative to cells cocultured with pSIN-expressing donor LEC. Columns are the average fold change from three independent co-culture assays; bars are the standard deviation from the mean. **, p<0.01,*, p<0.05 with respect to pSIN co-culture. (C) Heat map of the relative changes in expression of genes most significantly altered in LEC co-cultured with DLL4 compared to pSIN and the corresponding changes in cells receiving JAG1-induced signals. Gene probes are ordered according to the list highlighted in Table S1. (D) mRNA levels of cyclin F (CCNF), MCM10 and MCM4 in KLEC. Columns are the average of two independent experiments with respect to LEC. **, P<0.01 and *, P<0.05. (1.13 MB TIF) [file ppat.1000616.s004.tif]
