# Supplementary material for: KSHV Manipulates Notch Signaling by DLL4 and JAG1 to Alter Cell Cycle Genes in Lymphatic Endothelia
Source: PLoS Pathog. 2009 Oct 9;5(10):e1000616. doi: 10.1371/journal.ppat.1000616 (PMC2751827; doi:10.1371/journal.ppat.1000616)
Supplement: Protocol S1 — Supporting protocols. (0.02 MB PDF) [file ppat.1000616.s008.pdf]

Supporting Protocols

Cell Culture

LEC were obtained and verified as previously described [1] and cultured on fibronectin-coated flasks or wells in Endothelial Cell Growth Medium MV (PromoCell) supplemented with 10ng/mL of VEGF-C (R&D Systems). LEC were used for experiments at passages 3-6. Conditioned media from LEC or KLEC or pSIN- or vGPCR-expressing cells were obtained by incubating cells for 48 hours with media and passing the media through a 0.1μm filter to remove cell debris or virions. Conditioned media were either added to LEC or stored at –80°C.

BCBL1 cells containing a GFP–expressing recombinant KSHV [2] were cultured in RPMI 1640 (Invitrogen) containing 10% FBS and 400 ng/mL geneticin (Invitrogen). 293T cells were cultured in DMEM (Invitrogen) containing 10% FBS (Sigma), 100 units/mL penicillin G, and 100 μg/mL streptomycin (Invitrogen).

Cloning of pSIN/DLL4 and pSIN/JAG1

HUVEC total RNA was extracted and cDNA synthesis was performed as for LEC. HUVEC were cultured as described by the manufacturer (PromoCell). DLL4 sequence was cloned using PfuTurbo (Stratagene) according to the manufacturer's instructions and the following PCR conditions used: 95°C, 2min; 35 cycles of 95°C, 30sec; 52°C, 1min; 72°C, 7min; final extension, 72°C for 7min. JAG1 sequences were cloned using HotStar

HiFidelity Polymerase Kit (Qiagen) with Q Solution according to the manufacturer's instructions and the following PCR conditions used: 95°C 5min; 40 cycles of 94°C, 15sec; 54°C, 1min; 68°C 7min 30sec; final extension, 72°C for 10min.

Both DLL4 and JAG1 PCR reactions engineered a BamHI and NotI site at the 5' and 3' end of the sequence respectively. These sites were used to clone the sequences into the pSIN vector using restriction enzymes from Promega. Sequences were verified on an 3730XL Genetic Analyser using BigDye3.1 chemistry (Applied Biosystems) and cleaned up using the Agencourt CleanSeq System.

VEGFR Inhibition and VEGF Treatment of LEC

LEC were seeded at $1x10^5$ cells per well in six-well plates over night. Cells were treated with 50µM VEGFR inhibitor (Calbiochem) or the equivalent volume of DMSO for 6 hours before exposure to conditioned media from vGPCR-expressing or control LEC. RNA was collected after 18 hours. For VEGF treatment, cells were seeded as above and treated with 50ng/ml, 100ng/ml or 200ng/ml $VEGF_{165}$ (PromoKine). RNA was harvested after 6 hours and 24hrs.

References

1. Lagos D, Trotter MW, Vart RJ, Wang HW, Matthews NC, et al. (2007) Kaposi sarcoma herpesvirus-encoded vFLIP and vIRF1 regulate antigen presentation in lymphatic endothelial cells. Blood 109: 1550-1558.

2. Vieira J, O'Hearn P, Kimball L, Chandran B, Corey L (2001) Activation of Kaposi's sarcoma-associated herpesvirus (human herpesvirus 8) lytic replication by human cytomegalovirus. J Virol 75: 1378-1386.
